# Presence of insoluble Tau following rotenone exposure ameliorates basic pathways associated with neurodegeneration

**DOI:** 10.1016/j.ibror.2016.09.001

**Published:** 2016-09-26

**Authors:** Rodrigo S. Chaves, Amajad I. Kazi, Carolliny M. Silva, Michael F. Almeida, Raquel S. Lima, Daniel C. Carrettiero, Marilene Demasi, Merari F.R. Ferrari

**Affiliations:** a Department of Genetics and Evolutionary Biology – Institute for Biosciences, University of Sao Paulo, Sao Paulo, SP, Brazil; b Federal University of ABC, Sao Bernardo do Campo, SP, Brazil; c Laboratory of Biochemistry and Biophysics – Butantan Institute, Sao Paulo, SP, Brazil

**Keywords:** Hyperphosphorylated Tau, Hippocampus, Rotenone, Oxidative stress, Proteasome activity, Autophagy flux, Protein aggregation

## Abstract

Protein aggregation is an important feature of neurodegenerative disorders. In Alzheimer's disease (AD) protein aggregates are composed of hyperphosphorylated Tau and amyloid beta peptide (Aβ). Despite the involvement and identification of the molecular composition of these aggregates, their role in AD pathophysiology is not fully understood. However, depositions of these insoluble aggregates are typically reported as pathogenic and toxic for cell homeostasis. New evidences suggest that the deposition of these aggregates is a protective mechanism that preserves cell from toxic insults associated with the early stages of neurodegenerative diseases. To better understand the biological role of the protein aggregation with regard its effects in cellular homeostasis, the present study investigated the role of insoluble Tau and Tau aggregates on crucial cellular parameters such as redox homeostasis, proteasome activity and autophagy in hippocampal cell cultures and hippocampus of aged Lewis rats using a rotenone-induced aggregation model. Neurons were exposed to rotenone in different concentrations and exposure times aiming to determine the interval required for Tau aggregation. Our experimental design allowed us to demonstrate that rotenone exposure induces Tau hyperphosphorylation and aggregation in a concentration and time-dependent manner. Oxidative stress triggered by rotenone exposure was observed with the absence of Tau aggregates and was reduced or absent when Tau aggregates were present. This reduction of oxidative stress along with the presence of insoluble Tau was independent of alterations in antioxidant enzymes activities or cell death. In addition, rotenone induced oxidative stress was mainly associated with decrease in proteasome activity and autophagy flux. Conversely, when insoluble Tau appeared, autophagy turns to be overactivated while proteasome activity remained low. Our studies significantly advance the understanding that Tau aggregation might exert protective cellular effects, at least briefly, when neurons are facing neurodegeneration stimulus. We believe that our data add more complexity for the understanding of protein aggregation role in AD etiology.

## Highlights

• Rotenone triggers Tau hyperphosphorylation and aggregation in a time and concentration-dependent manner, being the temporal component predominant.
• Autophagy is modulated differently before and during Tau aggregation, while proteasome activity is reduced in both conditions.
• The formation of Tau aggregates normalizes cellular dysfunctions detected in the absence of them.

## Graphical abstract

**Figure:**
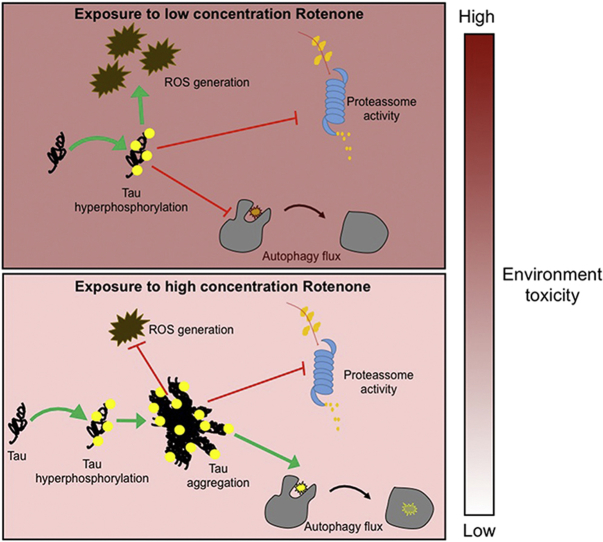

## 1 Introduction

Neurodegeneration is often associated with the presence of extra and intracellular protein aggregates distributed throughout the central nervous system (CNS). Several neurodegenerative disorders, including Alzheimer's disease (AD) and frontotemporal dementia, are characterized by the presence of intracellular inclusions of neurofibrillary tangles (NTFs) and paired helical filaments (PHFs). These inclusions are mainly composed of hyperphosphorylated Tau (Ross and Poirier, 2005, Frost et al., 2015).

Tau is a microtubule-associated phosphoprotein expressed primarily in the CNS and mainly distributed at the axonal compartment promoting microtubules assembly and stabilization. Abnormal hyperphosphorylation of Tau at various sites negatively regulates its function and is implicated in degeneration and neuronal loss in AD and other tauopathies. Hyperphosphorylation not only reduces Tau housekeeping functions but also increases its cellular toxicity where in hyperphosphorylated Tau sequester the normal Tau (Kuret et al., 2005) to form Tau oligomers, being these oligomers described as precursors of PHFs and NTFs (Alonso et al., 1996).

The presence of intracellular inclusions of Tau is believed to disrupt neuron physiology, through alterations in microtubule dynamics (Kuret et al., 2005), axonal transport (Cuchillo-Ibanez et al., 2008, Shahpasand et al., 2012, Smith and Bennett, 1997), synaptic function (Scheff et al., 2013) and loss of redox homeostasis (Melo et al., 2011). Proteasome and autophagy pathways, mechanisms involved in clearing hyperphosphorylated Tau/Tau aggregates, are also suggested to be dysregulated during the etiology of tauopathies (Huang and Figueiredo-Pereira, 2010, Ben-Gedalya and Cohen, 2012).

To date, several reports from animal and cellular models that recapitulate the basic stages of disease progression suggested that neuronal loss occurs before NFTs are formed. Tau oligomers that are formed during the early stages of protein aggregation are considered to be more toxic to cells than protein inclusions themselves (Chung et al., 2001, Flach et al., 2012, Hoover et al., 2010, Lasagna-Reeves et al., 2010). Although Tau is implicated in the pathology of AD, the contribution of few Tau species (oligomers and insoluble Tau) in disease initiation are not fully appreciated. Studies also report protective effects associated with the formation of protein inclusions in AD (de Calignon et al., 2010, Sydow et al., 2011) and other neurodegenerative diseases models (Bodner et al., 2006).

These findings emphasize the importance of studying cellular events that occur prior and during the formation of protein aggregation to advance our understanding of neurodegeneration process in AD. Thus, although protein aggregation has been extensively investigated, it biological role remains contradictory. We hypothesize that this conundrum rises with the fact that most reported studies have relayed on Tau overexpression or transgenic systems expressing mutant Tau, lacking endogenous regulation and possibly inducing extreme toxic environments distinctly from the environment induced by constitutive protein aggregation.

We showed previously that low concentrations (0.5–1 nM) of rotenone, a mitochondrial complex I inhibitor widely employed in studies related to neurodegeneration (Radad et al., 2008, Ullrich and Humpel, 2009), were able to induce hyperphosphorylation and aggregation of constitutive Tau in the absence of cell death in primary hippocampus cell cultures (Chaves et al., 2010). Chronic administration of rotenone to rats also induces cerebral tauopathy (Höglinger et al., 2005, Almeida et al., 2016) revealing rotenone as a reliable method to induce constitutive Tau aggregation in *in vivo* and *in vitro* systems.

Based on this, rotenone exposure could be used, as a system to indirectly evaluate whether dysfunctions in neuronal homeostasis occur before the formation of Tau aggregation and whether Tau aggregation influences positively or negatively these early dysfunctions.

Oxidative stress induced by increase in reactive oxygen species (ROS) is considered an early event occurring in AD, possibly acting as an inductor of protein aggregation (Tabner et al., 2005, Hands et al., 2011). In addition to oxidative stress, decrease in protein degradation pathways, proteasome and autophagy, have been reported during the initial stages of AD (Cecarini et al., 2007, Resende et al., 2008).

Proteasome system plays a pivotal role in clearing oxidized and misfolded proteins and inhibition of proteasome activity led to increase in Tau accumulation (Tseng et al., 2008) and hyperphosphorylation (Agholme et al., 2014, Carrettiero et al., 2009). Moreover, oxidative stress might impair protein degradation pathways, such as the proteasome system (Cecarini et al., 2007, Lam et al., 2000), and inhibition of proteasome activity can induce oxidative stress (Maharjan et al., 2014) demonstrating a cross-talk between both pathways in early phases of AD.

Since exposure to high doses of rotenone (100 nM) disrupt proteasome activity and increase ROS synthesis (Chou et al., 2010), and rotenone exposure also induce Tau hyperphosphorylation, it is plausible postulate that rotenone reproduce some early aspects related with AD pathophysiology. Here, we propose investigate, using hippocampal cell cultures and hippocampus of aged Lewis rats, (1) whether dysfunctions in redox homeostasis, protein degradation machinery and Tau hyperphosphorylation, triggered by exposure to rotenone, occur before Tau aggregation; and (2) whether dysfunctions induced by rotenone exposure in the absence of aggregates are potentiated or mitigated by the presence of insoluble Tau and Tau aggregates.

## 2 Experimental procedures

All the procedures were performed in strict accordance with Institutional and International Guidelines for animal care and use (Demers et al., 2006), as well as respecting the Brazilian federal law 11794/08 for animal welfare.

### 2.1 Primary neuronal cell culture and rotenone exposure

Methodology employed for cell culture was a modification of the previously described protocol (Kivell et al., 2001). Briefly, 20 neonatal (1 day-old) Lewis rats had their brains dissected out to access the hippocampus, which was dissociated in sterile cold solution consisting of 120 mM NaCl, 5 mM KCl, 1.2 mM KH_2_PO_4_, 1.2 mM MgSO_4_, 25 mM NaHCO_3_, 13 mM glucose, pH 7.2. Cell solution was centrifuged at 300 × *g* for 5 min. The supernatant was discarded and cells were suspended in Neurobasal A medium (Gibco) supplemented with 0.25 mM Glutamax (Gibco), 2% B27 (Gibco), 0.25 mM L-Glutamine (Sigma) and 40 mg/L Gentamicin (Gibco).

Cells were plated on 12-well nunclon (Nunc), 96-well plate (Nunc) or confocal dishes (MatTek), coated with poli-_D_-lysine, at the density of 1800 cells/mm^2^. Cultures were kept in a humidified incubator with 5% CO_2_ at 37 °C for nine days with the media changed every three days.

It was previously reported that exposure of primary cell cultures to rotenone in concentrations over 1 nM during 48 h induces massive cell death (Chaves et al., 2010). However, exposure to low concentration of rotenone (0.5 nM) during 48 h did not induce cell death and triggered Tau aggregation. Here, to further investigate rotenone effects in neuron homeostasis and in Tau aggregation, exposure to 0.5 and 0.3 nM of rotenone were used. Exposure to 0.3 nM was chosen to verify whether lowering rotenone concentration by 40% would impair its effects upon Tau aggregation.

Media were supplemented with rotenone (Sigma) 0.3 or 0.5 nM or with dimethyl sulfoxide (DMSO, Sigma), and applied to cell cultures during either 48 or 72 h. The concentration of DMSO was maintained at 0.002% in all treatment groups. Cells plated on confocal dishes were used for immunocytochemistry assays for MAP2 and Tau detection and to live cell analysis of ROS content and autophagy flux. Cells grown on 6 and 96 wells culture plates were used for western blot and biochemical assays.

### 2.2 Determination of cell viability by MTT reduction and trypan blue stain assays

MTT (3-(4,5-dimethylthiazol-2-yl)-2,5-diphenyltetrazolium bromide) assay was carried on 96 wells plated neuronal cultures. After each treatment, 20 μl of MTT (5 mg/ml) was added into each well and the plate was further incubated for 1 h at 37 °C. Media in each well was discarded and the colored products of MTT in cells were solubilized with 200 μl of DMSO. Optical intensity at 570 nm was read using Epoch, Biotek multiwell plate reader. Each group contained 6 independent measurements.

Trypan blue stain assay was performed adding 10 μL of Trypan blue stain solution (Gibco) to cultured cells media at the end of rotenone exposure time in order to stain in blue the cytoplasm of cells with damaged plasma membrane. Immediately after the addition of Trypan blue, cells were examined under an inverted microscope (Nikon Eclipse TS100) using a 10× objective (100× magnification) and brightfield images were acquired from three different fields randomly choose per plate per each experimental condition. Then, the number of Trypan blue positive cells per field was accessed.

### 2.3 Detergent soluble and insoluble protein fractions

Total protein samples were extracted in microtubule destabilizing conditions with modified RIPA buffer (50 mM Tris-HCl (pH 8.0), 150 mM NaCl, 1 mM EGTA, 1% Nonidet P-40, 0.25% sodium deoxycholate, 0.1% SDS, 0.5% Triton X-100, 1 mM 4-(2-aminoethyl) benzenesulfonyl fluoride hydrochloride, 1 μg/ml protease inhibitor cocktail (Sigma), 1 mM NaF, 1 mM Na_3_VO_4_), using homogenizer and followed by incubation at 4 °C for 30 min accordingly to a previously described method (Santa-Maria et al., 2007). Homogenates were centrifuged at 20,000 × *g*, 4 °C, 30 min. Supernatant were kept as soluble fraction and the pellet were washed twice with lysis buffer followed by centrifugation (20,000 × *g*, 4 °C, 30 min). The total amount of protein present in the insoluble pellet was extracted with equal volume of SDS sample buffer. Protein concentration from supernatant and insoluble fractions were determined using Bradford's reagent (Bradford, 1976).

### 2.4 Immunoblot assays for total Tau and hyperphosphorylated Tau detection

Proteins present in soluble and insoluble fraction were resolved on 10% SDS-polyacrylamide gels, transferred to nitrocellulose membrane and blocked (5% non-fat dry milk in TBS-T) during 1 h at room temperature, then probed with primary antibodies.

Membranes were incubated overnight at 4 °C with antibodies against hyperphosphorylated Tau (1:1000, Sigma, Ser 199/202, T6819, raised in rabbit), total Tau (1:1000, Sigma, T9450, raised in mouse), and β-actin (1:1000, Santa Cruz, sc-47778, raised in mouse). Membranes were washed with TBS-T (3X, 5 min) followed by incubation with HRP conjugated secondary antibodies (anti-rabbit 1:10000 (Amersham) or anti-mouse 1:6000 (Amersham)) during 1 h at room temperature. Blots were developed using ECL reagent according to manufacturers instructions (PerkinElmer).

### 2.5 Immunocytochemistry assays for hyperphosphorylated Tau and MAP2 detection

Neurons were fixed with methanol:acetone (1:1) for 10 min at −20 °C, rinsed with PBS and permeabilized with 0.2% triton X-100 in PBS for 30 min. Non specific binding was blocked with 2% normal goat serum and 4% BSA in PBS for 30 min and then incubated overnight with primary antibodies against hyperphosphorylated Tau (1:1000, Sigma, Ser 199/202, T6819, raised in rabbit) or MAP2 (1:1000, sc 74422; Santa Cruz, raised in mouse) in 1% NGS, 2% BSA and 0.2% triton X-100. Dishes were washed with cold PBS followed by incubation with FITC or Texas red conjugated secondary antibody for 2 h. Cells were mounted with mounting medium containing DAPI (4′,6-diamidino-2-phenylindole, Vector laboratories).

### 2.6 Proteasome activity

Proteasome activity was determined according to the previously described method (Silva et al., 2012). Cells and brain tissue in each treatment group were lysed in RIPA buffer, except for the absence of protease inhibitor cocktail. Thirty micrograms of total protein were incubated with 125 μM of the fluorogenic substrate s-LLVY-MCA (Calbiochem) in 20 mM Tris/HCl buffer, pH7.5, at 37 °C. This substrate is a standard peptide widely employed to specifically assess the chymotrypsin-like proteasome activity, independent of ubiquitination (Hanna et al., 2007). Fluorescence emission was recorded at 440 nm (excitation at 365 nm) for 45 min in Gemini XPS Fluorescence Microplate Reader.

### 2.7 Intracellular levels of ROS and hydrogen peroxide

Generation of ROS in live neurons was evaluated using the probe CM-H_2_DCFDA (Invitrogen), according to a previously described method (da-Silva et al., 2004). Briefly, ROS levels were assessed by incubating neurons 40 min with 2 μM of CM-H_2_DCFDA diluted in cell culture media, followed by two rinses with phenol free Neurobasal A medium (Invitrogen) and immediately subjected to live cell imaging analysis. Cells were imaged on Carl Zeiss LSM780 inverted Multiphoton microscope using an integrated live cell chamber allowing the maintenance of a 5% CO_2_ and 37 °C environment, images were acquired from 3 fields, randomly chosen, for each experimental condition. Experiment was performed in triplicates and the quantitative analysis performed using Image J (NIH) as described previously (Figueiredo et al., 2013).

To evaluate the intracellular H_2_O_2_ content a modification of Zhou et al. (1997) method was employed. Briefly, 30 μg of total protein extract of cell cultures or brain tissue were incubated with 50 μM of the fluorogenic reagent Amplex red (Molecular probes) and 1.0 U/mL of Horseradish peroxidase diluted in 0.1 M of phosphate buffer, pH 7.0, at 37° C. Fluorescence emission was recorded at 587 nm (excitation at 563 nm) for 10 min in Gemini XPS Fluorescence Microplate Reader.

### 2.8 Protein carbonylation

Protein carbonylation levels in soluble fraction were measured with Oxiselect Protein Carbonyl Immunoblot Kit (Cell Biolabs, Inc., San Diego, CA, U.S.). Fifteen micrograms of protein homogenates were resolved by SDS-polyacrylamide gel and transferred to nitrocellulose membranes. Membranes were then incubated with DNPH solution for derivatization accordingly to manufacturers instructions, followed by blocking during 1 h at room temperature in TBS-T with 5% non-fat dry milk. Blots were incubated with anti-β-actin antibody and developed as previously described for normalization.

Protein carbonylation was evaluated by incubating the membranes with primary antibody against DNP (1:1000, 230801, Cell Biolabs) in TBS-T with 5% non-fat dry milk during 1 h at room temperature. Membranes were washed in TBS-T and incubated with anti-goat horseradish peroxidase conjugated antibody diluted 1:1000 in solution containing 5% non-fat dry milk/TBS-T for 1 h at room temperature. Labeling was developed with enhanced chemiluminescence reagent and the whole smear of bands per gel lane considered for quantification. Quantification was done as already detailed, using β-actin for data normalization.

### 2.9 Antioxidant enzymes assays

Catalase activity in cell homogenates was assayed following the method described by Aebi (1984) by monitoring the decomposition of H_2_O_2_ (13 μM) in potassium phosphate buffer (50 mM, pH 7.0) at 240 nm. Enzyme units were expressed as μmoles H_2_O_2_ decomposed/min/mg protein.

Glutathione peroxidase was assayed using commercial Kit (Sigma) and enzyme activity was calculated based on the oxidation of glutathione (GSH) to oxidized glutathione (GSSG). This is catalyzed by Glutathione peroxidase coupled to the recycling of GSSG back to GSH utilizing glutathione reductase and NADPH. The decrease in NADPH absorbance measured at 340 nm during the oxidation of NADPH to NADP is indicative of glutathione peroxidase activity since the enzyme is the rate-limiting factor of the coupled reactions. Results are expressed as μmoles of oxidized NADPH/mg protein/min.

### 2.10 Autophagy flux

Autophagy flux was monitored expressing the LC3-eGFP-mCherry construct (Kabeya et al., 2000, Kimura et al., 2007). The expression of eGFP is restricted to autophagosomes and the mCherry labeling can be found at the autophagosomes and autophagolysosomes compartments. Autophagy flux is the ratio between the number of vesicles presenting both fluorescence emitted by eGFP and mCherry, and the vesicles presenting only mCherry signal (Castillo et al., 2013, Kimura et al., 2007, Nyfeler et al., 2011).

After 8 days of culture, cells were transfected with 1750 ng of LC3-EGFP-mCherry using lipofectamine 2000 (Invitrogen), according to manufacturer's protocol, followed by 48 h of 0.3 or 0.5 nM of rotenone exposure using phenol-free Neurobasal A culture medium. Three cells per dish from 5 different cultures were evaluated at excitation wavelengths of 488 nm for EGFP and 535 nm for mCherry, and emission 520 nm and 620 nm, respectively. Cells were imaged on Carl Zeiss LSM780 inverted Multiphoton microscope using an integrated live cell chamber allowing the maintenance of a 5% CO2 and 37 °C environment. Quantitative analysis of immunofluorescence data was performed using Image J (NIH) and the plug-in *Green and Red puncta colocalization* as described previously (Pampliega et al., 2013). Autophagic flux was accessed according to Castillo and colleagues' protocol (Castillo et al., 2013).

Autophagy was also monitored by western blot assays evaluating LC3 II and Beclin-1 protein levels. Briefly, western blot assays were performed similarly to previously described herein, membranes were incubated for 1 h at room temperature with anti-beclin-1 (1:500, Santa Cruz, sc-11427, raised in rabbit) or overnight with anti-LC3II (1/1000, Sigma L8918, raised in rabbit) and developed as stated before.

### 2.11 Protein aggregation and oxidative stress in aged rats

Aged (12 months old) Lewis rats were anaesthetized with ketamine and xylazine for subcutaneous insertion of osmotic minipumps (Alzet). Minipumps were filled either with rotenone dissolved in equal volumes of DMSO and polyethylene glycol (PEG, Sigma, USA) which was delivered at the rate of 1 mg/kg/day or 2 mg/kg/day during 4 weeks, or only DMSO:PEG (1:1) as control. After treatment animals were euthanized and their hippocampus removed to analyze hyperphosphorylated and total Tau levels, proteasome activity and intracellular hydrogen peroxide content.

For immunocytochemistry, rats were deeply anaesthetized and perfused with saline (0.9%) followed by a fixative solution consisted of 4% paraformaldehyde (w/v) and 0.2% picric acid (v/v) in 0.1 M PBS, pH 6.9, at 4 °C. Hippocampus was removed and fixed for additional 90 min in fixative solution described above, following incubation in sucrose diluted in PBS for 48 h, sucrose was changed every 24 h. Brain sections (14 μm) were obtained in a cryostat (Leica, CM3050, Germany), at −25 °C. Sections were incubated for 24 h at 4 °C with antibody against hyperphosphorylated Tau 1:200 (Santa Cruz; sc101813), followed by incubation with biotinylated secondary antibody goat anti-rabbit immunoglobulin (1:230, Vector, USA) for 90 min. Sections were next incubated with avidin-biotin peroxidase complex (1:120, Vectastain, Vector, USA) for 45 min and immunoreactivity was visualized using 3-3′-diaminobenzidine tetrahydrochloride (DAB, Sigma) as chromogen and H_2_O_2_ (0.05%) during 6 min of reaction. Following DAB reaction cells were counterstained with hematoxylin and examined under microscope (Nikon Eclipse TS100) using 10× and 20× objectives, images were acquired from three different fields randomly choose per plate per each experimental condition for evaluation of the staining profile.

Hippocampus from aged rats exposed to 1 mg/kg/day of rotenone or DMSO:PEG as the control, was removed for protein extraction in order to evaluate hyperphosphorylation of Tau, proteasome activity and H_2_O_2_ content, using the same methodology as described earlier for cultured hippocampal cells.

### 2.12 Statistical analysis

Results were evaluated by one-way ANOVA followed by Tukey post-test for cell culture analysis and Student's T-test for aged rat. Statistics was assessed through GraphPad Prism (GraphPad Software Inc., version 4.00, CA). A p-value ≤0.05 was considered to indicate statistically significant differences. It was employed a minimum of n = 3 for cell cultures and n = 5 for animals. All experiments were repeated at least twice and performed in technical triplicates.

## 3 Results

### 3.1 Rotenone concentration and exposure time dictates whether Tau hyperphosphorylation is accompanied or not by Tau aggregation

**Fig. 1:**
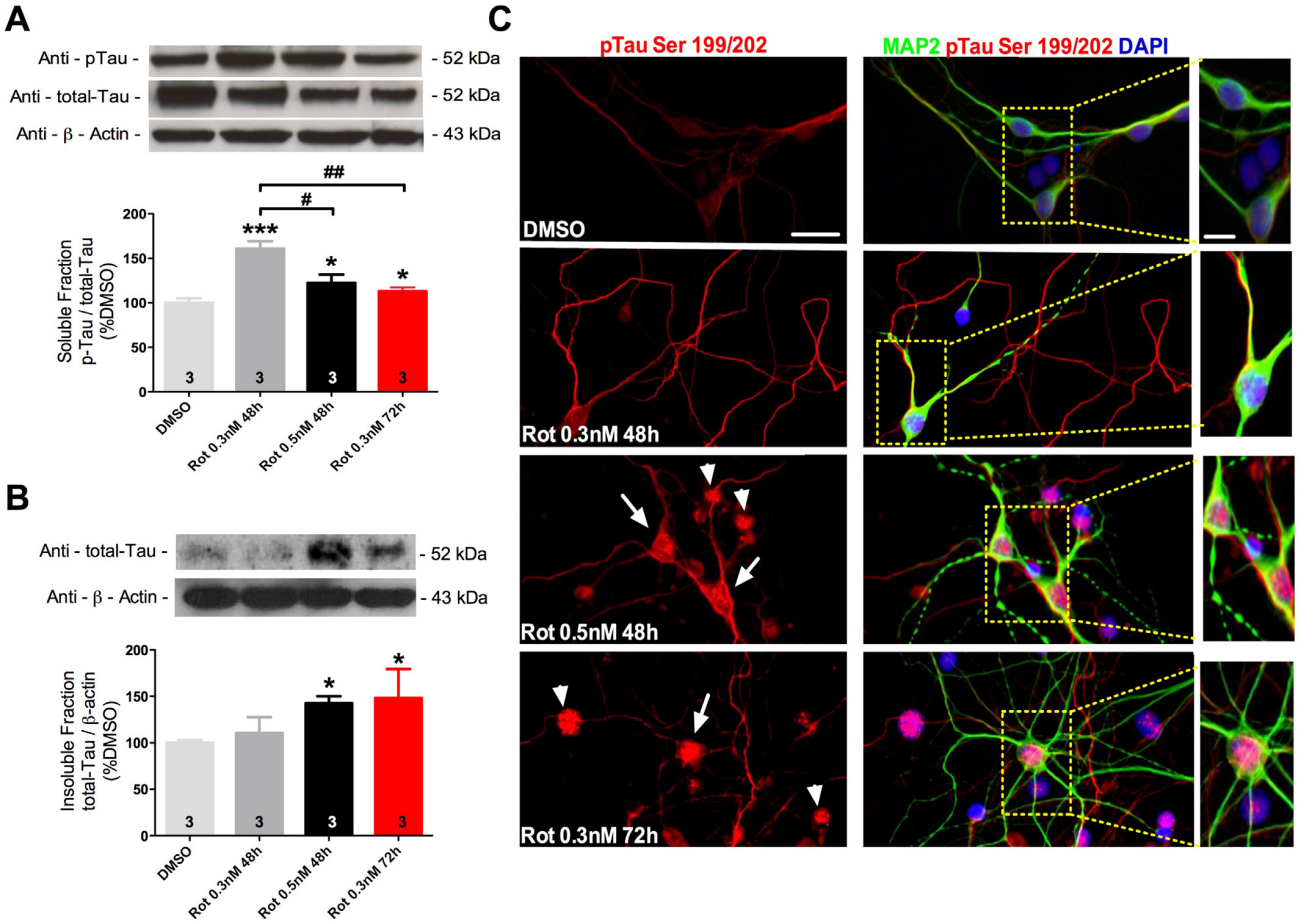
Quantitative analysis of Tau levels in cultured hippocampal cells exposed to DMSO, 0.3 or 0.5 nM of rotenone during 48 or 72 h. Hyperphosphorylated Tau normalized to total-Tau in protein soluble fraction (A). Total Tau normalized to β-actin in protein insoluble fraction (B). Illustrative digital images of cultured hippocampal neurons labeled with antibodies against hyperphosphorylated Tau (p-Tau) and MAP2 after exposure to rotenone (C). Dense p-Tau aggregates are found in neuronal (arrows) and non-neuronal (arrow heads) cells exposed to 0.5 nM of rotenone during 48 h, as well as 0.3 nM of rotenone after 72 h. Values are shown as percent control (DMSO) ± S.D obtained from three independent experiments. *p < 0.05, ***p < 0.001 as compared to DMSO; #p < 0.05, ##p < 0.01 compared as indicated, according to one-way ANOVA followed by Bonferroni ad-hoc test. Scale bar = 50 μm for low magnification and 10 μm for high magnification pictures in (C).

Since Tau hyperphosphorylation is a crucial step for Tau aggregation we quantitatively measured Tau hyperphosphorylated levels. Soluble levels of hyperphosphorylated Tau normalized to total Tau levels in hippocampal cultures revealed an increase by 60% and 35%, 48 h after exposure to 0.3 nM and 0.5 nM of rotenone, respectively, as compared to DMSO (Fig. 1A).

To evaluate whether the observed increase in Tau hyperphosphorylated levels was sufficient to trigger Tau aggregation, we performed experiments measuring total Tau levels in detergent insoluble protein fraction and immunocytochemistry assays to detect Tau aggregation. Insoluble fraction analysis showed a significant increase by 45% in total Tau levels following exposure to 0.5 nM of rotenone during 48 h, strikingly exposure to 0.3 nM of rotenone during the same period did not induce increase in Tau total levels in the insoluble fraction (Fig. 1B).

Immunocytochemical labeling of hippocampal cultures showed that immunoreactivity for Tau hyperphosphorylated is mainly localized in the neurites and its raises as rotenone concentration increase (Fig. 1C). In cells exposed to 0.5 nM of rotenone Tau hyperphosphorylated staining tend to mainly localize towards the cell body, illustrated by the increase in co-localization between MAP2 and hyperphosphorylated Tau stains, suggesting Tau aggregation in this cellular compartment (Fig. 1C).

Interestingly, DMSO exposed cells displayed smooth and elongated labeling for microtubule associated protein 2 (MAP2), although cell exposed to rotenone displayed puncta labeling, probably indicating microtubule fragmentation triggered by Tau hyperphosphorylation. Hyperphosphorylated Tau was also detected in several non-neuronal cells (MAP2 negative) exposed to 0.5 nM of rotenone during 48 h (Fig. 1C).

Since the exposure to 0.3 nM of rotenone during 48 h induced Tau hyperphosphorylation without triggering Tau aggregation, we hypothesize that rotenone effect upon Tau aggregation in your system could be also time-dependent. To evaluate our hypothesis hippocampal cell cultures were exposed to 0.3 nM of rotenone during 72 h and Tau hyperphosphorylation and aggregation were evaluated.

Compared to exposure to 0.3 nM of rotenone during 48 h, exposure for 72 h induced a 30% decrease in Tau hyperphosphorylated levels in the soluble fraction (Fig. 1A), being this decrease accompanied by a 20% increase in total Tau levels in the insoluble fraction (Fig. 1B). Immunocytochemistry assays in cells exposed to 0.3 nM rotenone during 72 h demonstrated a clear shift of Tau hyperphosphorylated staining from neurites only, as observed in cells exposed to 0.3 nM of rotenone during 48 h and controls, towards to a massive staining in neurons cell bodies and other non-neuronal cells illustrating Tau aggregation.

**Fig. 2:**
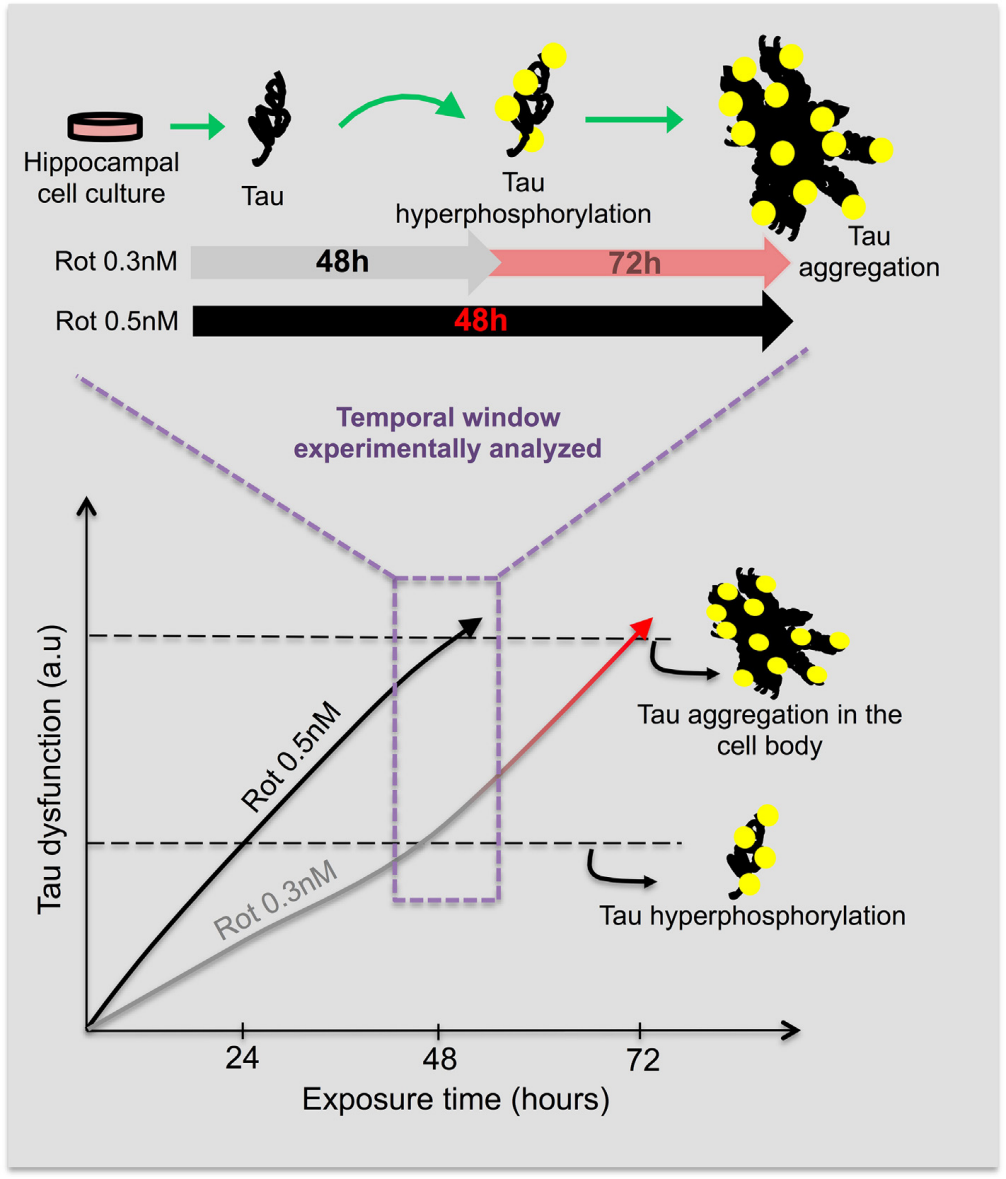
Scheme illustrating time and dose-dependent effects of rotenone exposure over tau dysfunctions in hippocampal cell cultures. The exposure to 0.3 nM of rotenone during 48 h induces Tau hyperphosphorylation without triggering Tau aggregation, however at longer timer point (72 h) Tau aggregation is present, suggesting that the earlier dysfunction observed at 48 h precedes and might trigger Tau aggregation at 72 h. Exposure to 0.5 nM of rotenone during 48 h triggers both dysfunctions in Tau homeostasis, hyperphosphorylation and aggregation, however faster than exposure to 0.3 nM of rotenone.

Thus, rotenone triggers Tau hyperphosphorylation and aggregation in a time and concentration-dependent manner (Fig. 1). Accordingly with our findings we postulate that the concentration and time dependence for the formation of Tau aggregation found in our model, allow us to mimic two different scenarios of Tau dysfunction in the cultured neurons (Fig. 2).

The first scenario is induced after exposure to 0.3 nM of rotenone during 48 h and occurs before Tau aggregation, where only Tau hyperphosphorylation is present in the cultured hippocampal neurons. Since exposure to 0.3 nM of rotenone during 72 h triggers Tau aggregation, this suggests that the early dysfunction observed at 48 h precede and might trigger Tau aggregation at 72 h.

In the second scenario, during Tau aggregation, Tau hyperphosphorylation is accompanied with the formation of Tau aggregates, being this scenario induced after exposure to 0.5 nM of rotenone during 48 h or 0.3 nM of rotenone during 72 h. The fact of Tau hyperphosphorylation levels, insoluble total Tau levels and immunocytochemistry analysis did not differ between primary cell cultures exposed to 0.5 nM of rotenone during 48 h or 0.3 nM of rotenone during 72 h further support our rationale.

### 3.2 Tau aggregation decreases the initial dysfunction in ROS levels promoted by soluble hyperphosphorylated Tau

To validate our rationale we analyzed ROS synthesis in both hypothetical scenarios. Oxidative stress is reported as a general inductor of protein aggregation and the aggregates themselves are suggested as additional generators of ROS.

**Fig. 3:**
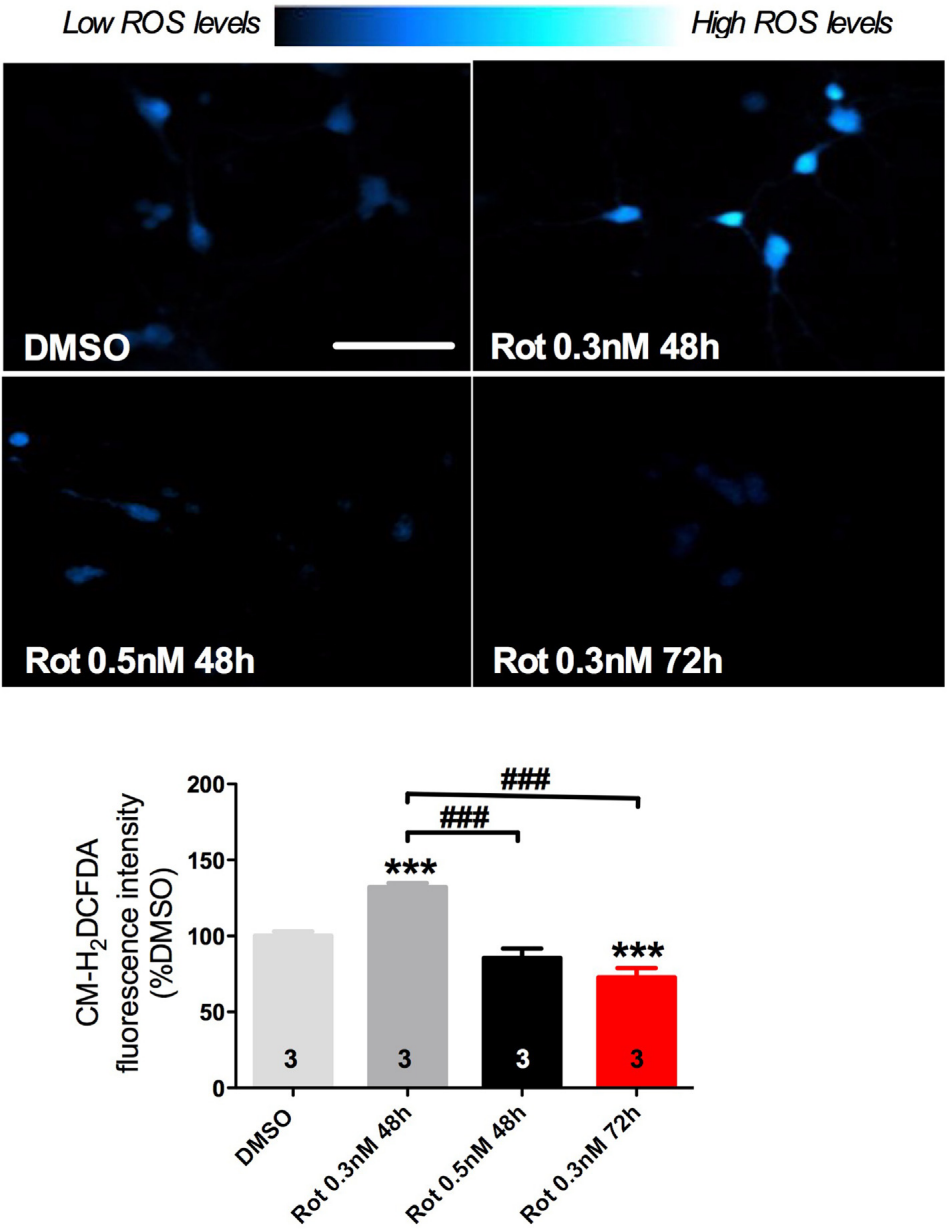
Illustrative confocal images of CM-H_2_-DCFDA staining in hippocampal primary cell cultures after rotenone exposure in the absence (0.3 nM during 48 h) and presence of Tau aggregates (0.5 nM during 48 h and 0.3 nM during 72 h), and quantitation of CM-H_2_-DCFDA fluorescence intensity among experimental groups. Values are shown as percent control (DMSO) ± S.D obtained from three independent experiments. *p < 0.05, ***p < 0.001 as compared to DMSO; #p < 0.05, ###p < 0.001 compared as indicated, according to one-way ANOVA followed by Bonferroni ad-hoc test. Scale bar = 50 μm.

In your model ROS content was significantly higher by 40% after exposure to 0.3 nM of rotenone during 48 h while in the presence of protein aggregation (0.5 nM for 48 h or 0.3 nM for 72 h) we found a significant decrease in cellular ROS content as compared with the scenario before Tau aggregation (Fig. 3). As expected, ROS level was similar between primary cell cultures exposed to 0.5 nM of rotenone during 48 h or 0.3 nM of rotenone during 72 h, illustrating the reliability of our system.

Striking, our results suggest that the formation of Tau aggregates decrease the initially increased ROS levels promoted by rotenone exposure and provide evidences that ROS dysfunction occurs even before the formation of Tau aggregates.

### 3.3 Tau aggregation effects in oxidative stress are uncoupled from alterations in the activity of antioxidant enzymes or cell death

Rotenone by definition is a classic inductor of oxidative stress, thus we aimed to further verify if the presence of Tau aggregates restores or mitigates rotenone effects in other parameters of neurons redox homeostasis. Since the exposure to 0.5 nM of rotenone during 48 h triggered Tau aggregation at the same levels that 0.3 nM during 72 h, we chose to use the exposure to 0.5 nM of rotenone during 48 h for the following analyses.

**Fig. 4:**
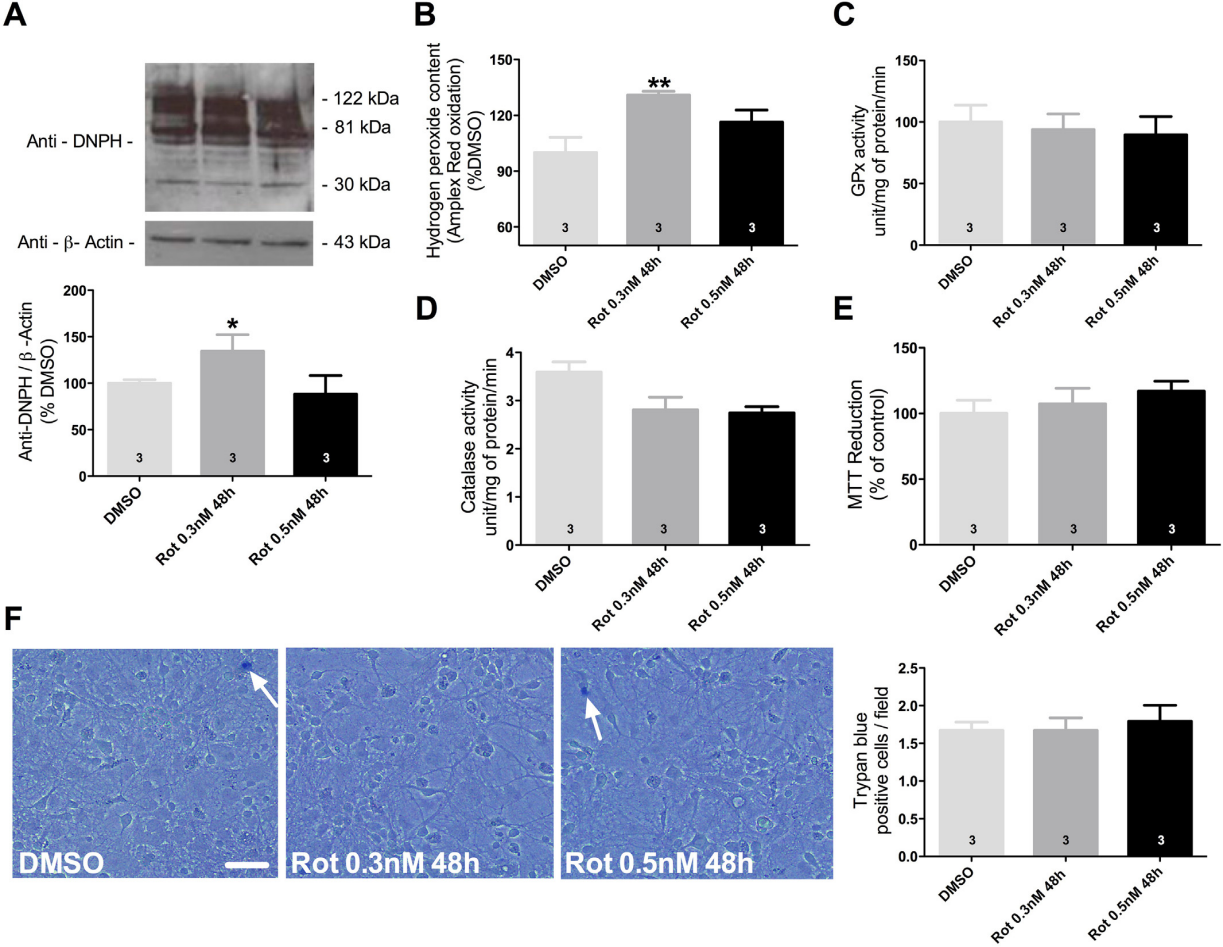
Oxidative stress and cell viability after rotenone exposure in the absence (0.3 nM during 48 h) and presence of Tau aggregates (0.5 nM during 48 h). Protein carbonyls levels after exposure to rotenone, whole lanes were considered for density quantification (A). Measurement of H_2_O_2_ levels (B) and Glutathione peroxidase (GPx) (C) and Catalase (D) enzymatic activity. Cell viability was evaluated by MTT reduction (E) and Trypan blue labeling (F). Data are shown as percent of control (DMSO) ± S.D. or mean ± S.D. obtained from three independent experiments. *p < 0.05; **p < 0.01 as compared to DMSO according to one-way ANOVA followed by Bonferroni ad-hoc test. Scale bar = 100 μm.

Protein carbonylation is a standard marker of oxidative stress, evaluation of rotenone effect upon protein carbonylation levels demonstrated an increase of 35% in neuronal cultures after exposure to 0.3 nM as compared with DMSO-exposed cells, while in the presence of Tau aggregates no significant difference was found (Fig. 4A). H_2_O_2_ is a classical upstream inductor of ROS, analysis of H_2_O_2_ levels in our model detected that exposure to 0.3 nM of rotenone increased H_2_O_2_ by 30% as compared to DMSO exposed cells, supporting our previous findings with protein carbonylation levels, the presence of Tau aggregates seems to mitigate rotenone effects (Fig. 4B).

Together, our data strongly suggest that the presence of Tau aggregates mitigated redox dysfunctions associated with rotenone exposure in absence of Tau aggregates. We hypothesize that alteration in the activity of antioxidant enzymes could be related with our findings. Evaluation of antioxidant enzymes activity, mainly glutathione peroxidase and catalase, demonstrated that enzymatic activity remained unchanged after exposure to rotenone compared with DMSO treated group (Fig. 4C and D). These findings suggest that antioxidant enzymes are not induced to cope up with cellular ROS generated following rotenone exposure or during Tau aggregation.

Cell death assays were performed to role out whether the identified decrease in redox homeostasis parameters was associated with cell death or decrease in cell viability. Both, MTT and Trypan blue labeling assays illustrated the absence of cell death followed by rotenone exposure associated or not with the presence of Tau aggregates, suggesting that cell death is not associated with the decrease of ROS levels related with rotenone effect in the presence of Tau aggregates (Fig. 4E and F).

### 3.4 Decreased autophagy flux triggered by rotenone is rescued by the presence of Tau aggregates

We hypothesized that the lack of ROS induction in the presence of Tau aggregates could be related with an up-regulation in the activity of protein degradation pathways such as autophagy and proteasome.

Impairment of protein degradation pathways is suggested as crucial towards the formation of protein aggegates and the artificial activation of these pathways can delay or even repair dysfunctions induced by protein aggregates in neurodegenerative diseases. To investigate the role of autophagy in the formation of Tau aggregates in our system, we evaluated autophagy flux after exposure to rotenone in the absence and presence of Tau aggregates.

**Fig. 5:**
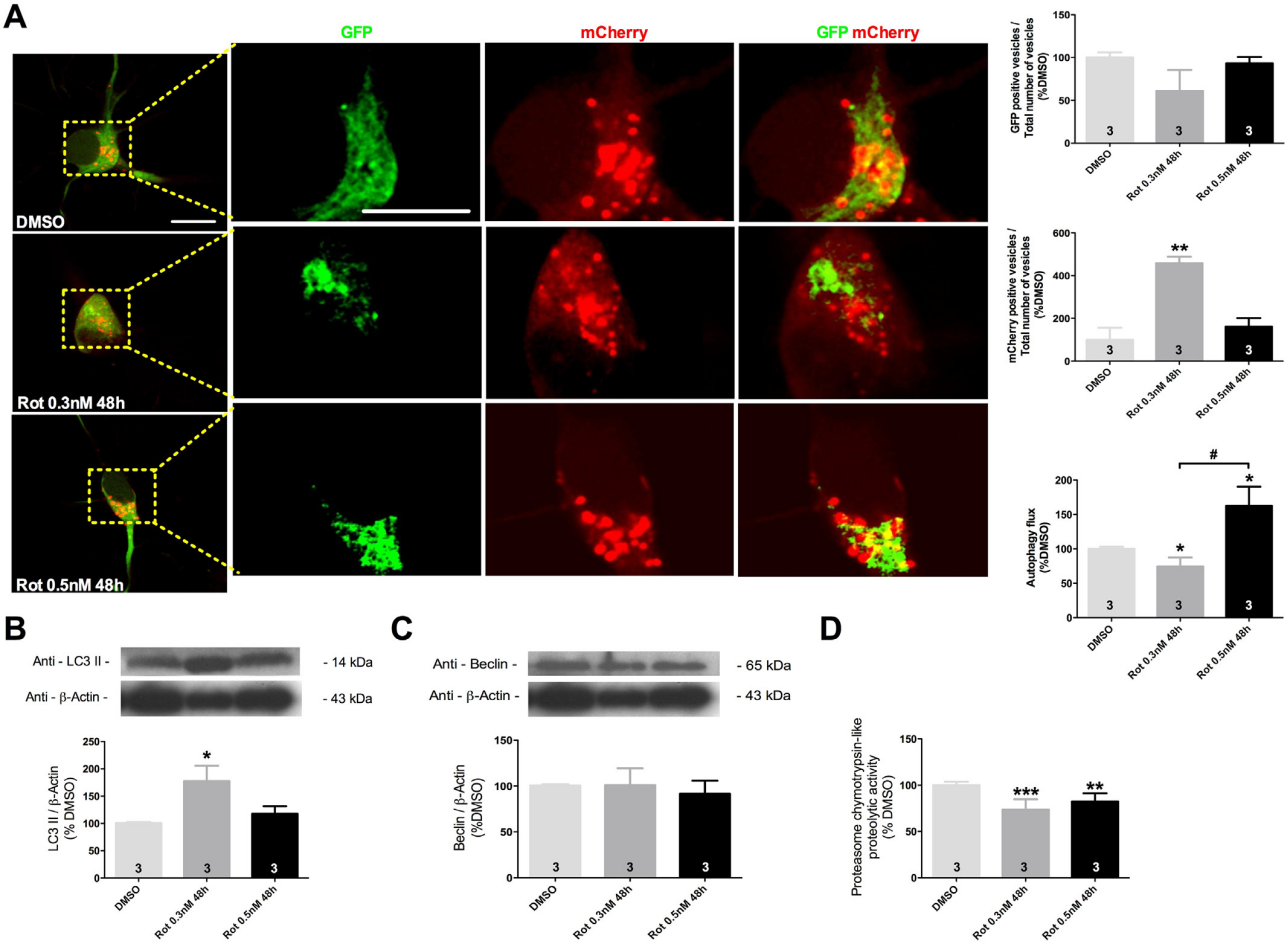
Autophagy flux and proteasome activity in hippocampal cultures after rotenone exposure in the absence (0.3 nM during 48 h) and in presence of Tau aggregates (0.5 nM during 48 h). Illustrative confocal images of LC3-eGFP-mCherry expression in hippocampal neurons following DMSO or rotenone exposure. Quantification of GFP and mCherry positive vesicles per the total amount of vesicle identified per cell and the evaluation of the autophagy flux in LC3-eGFP-mCherry transfected neurons (A). LC3II (B) and Beclin-1 (C) levels normalized to beta-actin in culture cells exposed to DMSO or rotenone. Proteasome activity analysis after exposure to DMSO or rotenone (D). Values are shown as percent control (DMSO) ± S.D obtained from three independent experiments. *p < 0.05; **p < 0.01; ***p < 0.001; as compared to DMSO according to one-way ANOVA followed by Bonferroni ad-hoc test. Scale bar = 30 μm.

Hippocampal cells exposed to 0.3 nM of rotenone during 48 h illustrated an increase in the number of autophagolysosomes vesicles without detectable changes in the number of autophagosomes vesicles. The analysis of autophagic flux in these cells revealed a 20% decrease in autophagic flux, supporting the increase in the number of autophagolysosomes vesicles and demonstrating a reduction in autophagy process (Fig. 5A). Meanwhile, in the presence of Tau aggregates rotenone exposure did not alter the number of autophagolysosomes or autophagosomes vesicles but did induce a massive increase in autophagy flux (Fig. 5A).

To determine how autophagy is affected, we evaluated the levels of LC3 II and Beclin-1 key proteins involved in autophagy process by western blot. Exposure to 0.3 nM of rotenone for 48 h induced 40% increase in LC3 II levels indicating failure in the autophagy process since increases in LC3II indicate accumulation of autophagolysosomes and autophagosomes, exposure to 0.5 nM did not induce changes in LC3 II levels (Fig. 5B). Beclin-1 expression levels before and during Tau aggregation did not show alteration (Fig. 5C), suggesting that nucleation of autophagic vesicles seems to be preserved during protein aggregation, and reinforcing that fusion of autophagic vesicles and lysosomes might be impaired.

We complemented our analysis of degradation pathways evaluating proteasome proteolytic activity. Exposure to rotenone independently of the presence or absence of Tau aggregates significantly reduced proteasome activity by 20% and 10%, respectively when compared with DMSO-exposed cells (Fig. 5D).

**Fig. 6:**
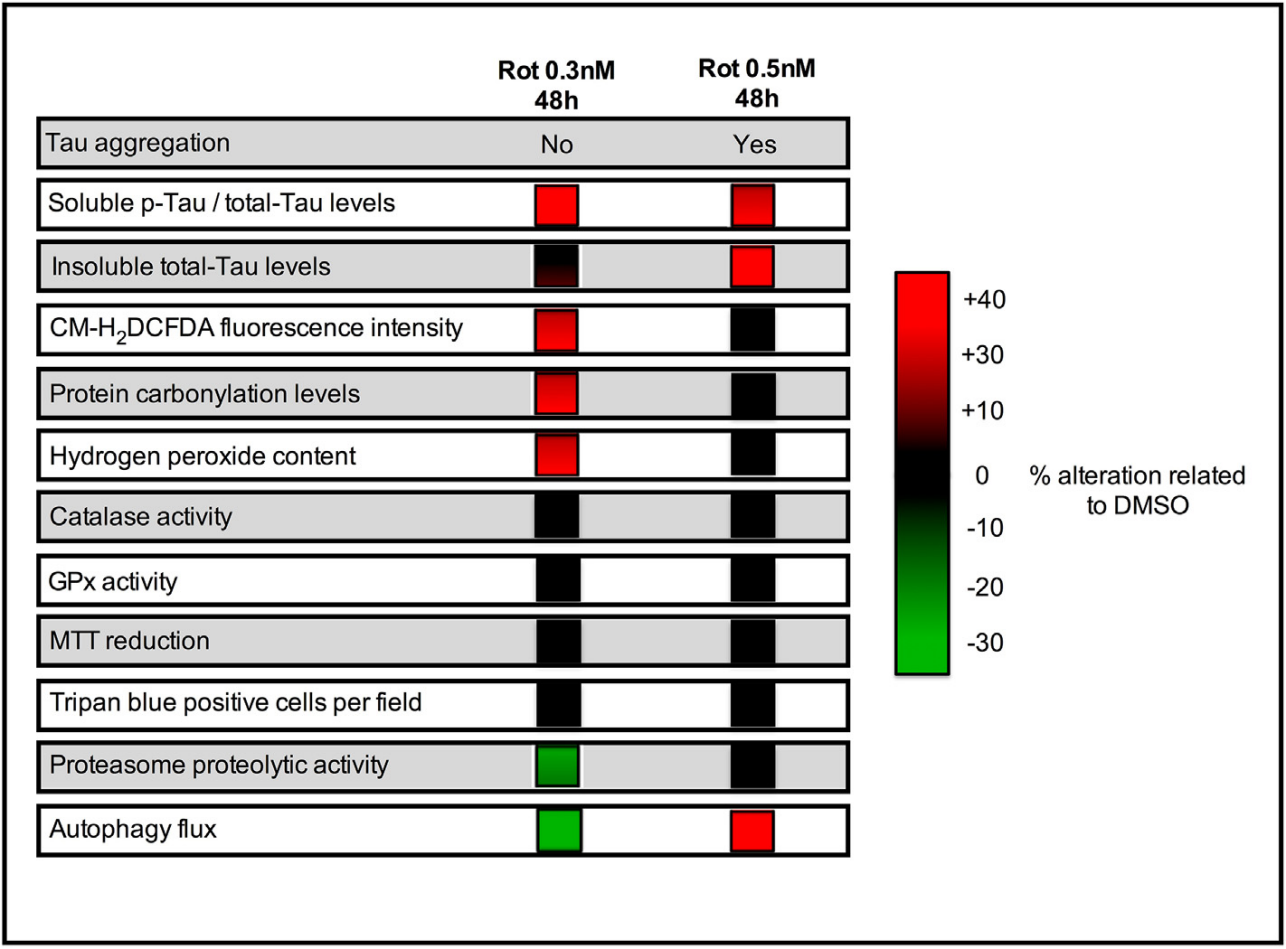
Summary of results achieved in the present study. Note that the formation of Tau aggregates mitigates most dysfunctions initially triggered by rotenone exposure. Rot: Rotenone, p-Tau; Hyperphosphorylated and GPx: Glutathione peroxidase.

Thus, we identified that autophagy is modulated differently when rotenone exposure induces Tau aggregates than when it does not, while proteasome activity is reduced in both conditions. Interestingly, our data suggest that the formation of Tau aggregates normalizes or mitigates cellular dysfunctions detected in the absence of them (Fig. 6), suggesting a possible protective effect associated with the formation of Tau aggregates.

### 3.5 H_2_O_2_ levels and proteasome activity are disrupted independently of Tau aggregation in the hippocampus of aged rats

Rotenone can also promote Tau hyperphosphorylation and aggregation in a time and concentration- dependent manner in aged rats resembling our experimental protocol for hippocampal cell cultures (Almeida et al., 2016). Then to further expand our findings, we evaluated whether parameters such as H_2_O_2_ levels and proteasome activity are also disrupted before the formation of Tau aggregates in the hippocampus of aged rats.

**Fig. 7:**
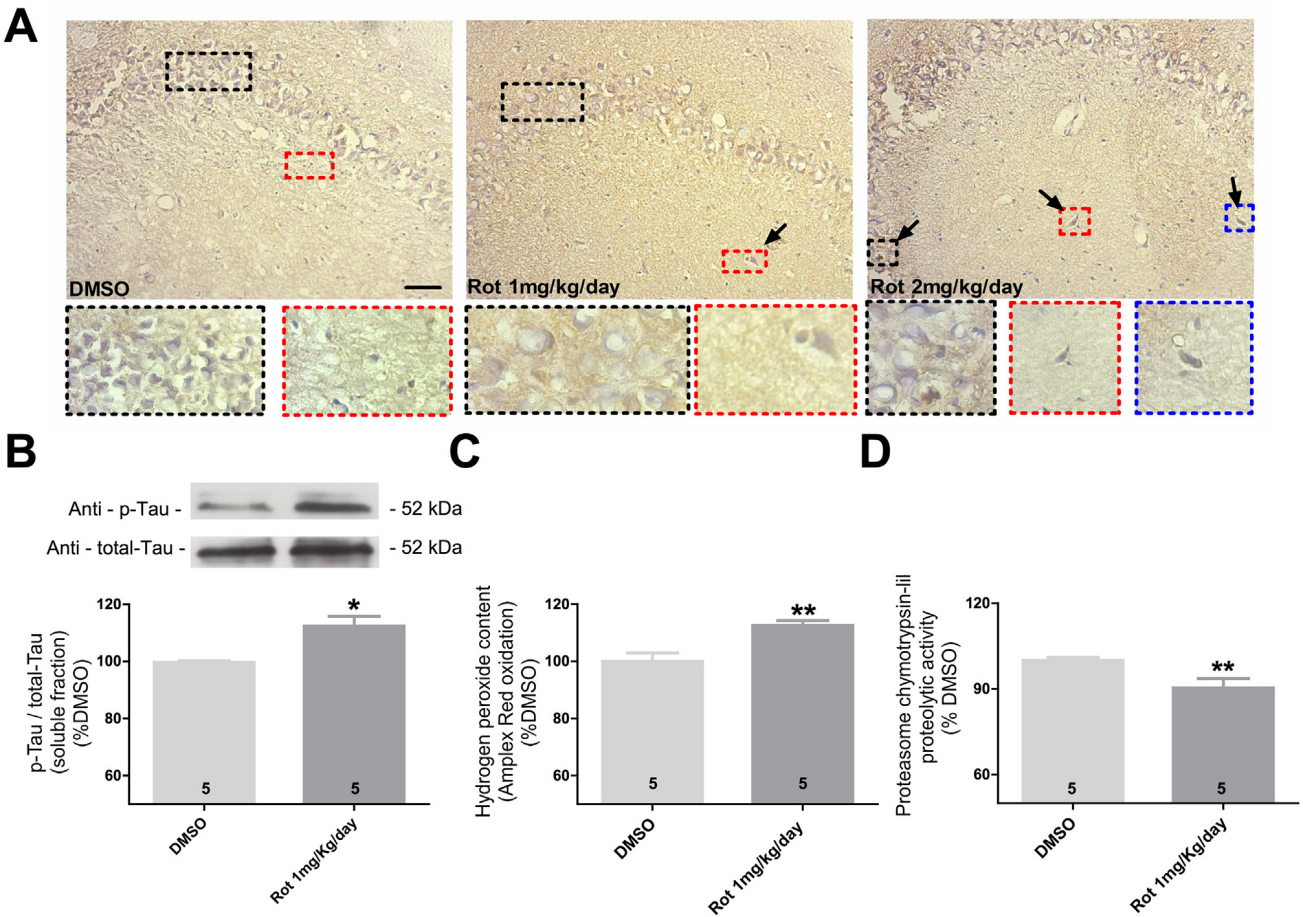
Tau hyperphosphorylation, Tau aggregation, H_2_O_2_ levels and proteasome activity in the hippocampus of aged rats after rotenone exposure. Representative digital images showing hyperphosphorylated Tau immunoreactivity and illustrating Tau inclusions after exposure to 2 mg/kg/day of rotenone (A). Quantitative analysis of hyperphosphorylated Tau (p-Tau) levels normalized to total-Tau in the hippocampus of aged rats exposed to DMSO or 1 mg/kg/day (B). Proteasome activity (D) and measurement of H_2_O_2_ levels (E) in the hippocampus of aged rats exposed to rotenone in the absence of Tau aggregates. Data are shown as percent of control (DMSO) ± S.D.; n = 5 for each experimental group; **p < 0.01 as compared to DMSO according to Student's T-test. Scale bar = 100 μm.

Aged rats exposed to 1 mg/kg/day of rotenone did not show intracellular hyperphosphorylated Tau inclusions in hippocampus, however with exposure to 2 mg/kg/day of rotenone we identified dense intracellular hyperphosphorylated Tau immunolabeling (Fig. 7A), although we were not able to infer about the morphology or distribution of this aggregates inside of stained cells. Accordingly with this result we selected the exposure to 1 mg/kg/day of rotenone as possible candidate to evaluate parameters influenced by rotenone exposure in the absence of Tau aggregates.

As expected, exposure to 1 mg/kg/day induced 10% increase in Tau hyperphosphorylated levels in hippocampus as compared to age-matched DMSO-exposed rats (Fig. 7B), illustrating Tau dysfunction independently of the presence of aggregates. In the absence of Tau aggregates, these aged rats also presented a significant decrease of 10% in proteasome activity and an increase, of the same magnitude, in H_2_O_2_ levels (Fig. 7C and D, respectively).

Identify the same pattern of dysfunction observed *in vitro* using an *in vivo* model strengths our results and suggests that the same array of rotenone induced dysfunctions that trigger Tau aggregation *in vitro* might happen *in vivo* as well.

## 4 Discussion

Here we have shown that exposure to rotenone promotes Tau hyperphosphorylation and aggregation, dysfunctions in redox homeostasis and decrease in the activity of protein degradation pathways, all in the absence of cell death and before it triggers Tau aggregation. We also found that the formation of Tau aggregates mitigates most dysfunctions initially triggered by rotenone exposure, suggesting that Tau aggregation might exert protective cellular effects, at least briefly, when neurons are facing neurodegeneration stimulus.

In our previous study, we showed that exposure to rotenone (1 nM) can induce Aβ and alpha-synuclein aggregation in hippocampal cell cultures, however Tau aggregation required 50% less rotenone to be triggered (0.5 nM), suggesting that rotenone effect upon Tau aggregation is stronger and take place early than Aβ and α-synuclein aggregation. Furthermore, our group also found that in hippocampus of aged Lewis rats, exposed to 2 mg/kg/day of rotenone during 4 weeks, Tau hyperphosphorylation and aggregation is detected in the absence of α-synuclein dysfunction supporting our data (Almeida et al., 2016). In this study, we extend our understanding of Tau pathology analyzing the interplay between oxidative stress and protein degradation pathways during the progression of Tau aggregation.

Events that precede cellular loss are important for determining disease etiology. In this study, results from *in vivo* and *in vitro* model suggest that early cellular alterations related with neurodegenerative processes occur earlier than Tau aggregation. Although, the real cause and consequence of protein aggregation in neurodegenerative diseases remains controversial (Congdon and Duff, 2008), the presence of intracellular Tau aggregates may be of relevance during neurodegenerative disease pathophysiology (Flach et al., 2012).

Tau hyperphosphorylation is considered the primary event in Tau dysfunction in AD progression. In this study we found that, exposure to low concentration of rotenone (0.3 and 0.5 nM) increased Tau hyperphosphorylation in hippocampal neurons as analyzed by antibody specific for Ser199/202 epitopes through immunocytochemistry and immunoblot assays. Activation of many kinases can trigger Tau phosphorylation of which glycogen synthase kinase-3β (GSK3β), MAPK and JNK have been shown to play a part in AD progression (Pei et al., 2001).

Rotenone induced hyperphosphorylation of Tau is suggested to be induced by the activation of GSK3β (Hongo et al., 2012) and occurs at a large number of phosphorylation sites (Ser 396, S202/T205, T212/S214 and S396/S404) (Han et al., 2014, Höglinger et al., 2005). These findings support our credence of using rotenone at low concentration as a model to study Tau pathology.

In the presents study, although increased hyperphosphorylated Tau was evident following exposure to 0.3 and 0.5 nM of rotenone for 48 h, Tau aggregates in the insoluble fraction were only identified in cells exposed to 0.5 nM of rotenone. However, neuronal cultures exposed to 0.3 nM of rotenone for prolonged period (72 h) also displayed Tau aggregation, suggesting that the earlier dysfunctions observed (at 48 h) might trigger Tau aggregation at later time points (72 h). Our findings suggested that we could take advantage of rotenone's effect been dose and time-dependent to evaluate whether the formation of Tau aggregates could further increase dysfunctions already triggered by rotenone exposure.

Thus, since the occurrence of ROS dysfunctions following rotenone exposure is well described (Dukes et al., 2005, Sherer et al., 2003) and evidences suggest that accumulation of insoluble and phosphorylated Tau in neurodegenerative diseases is a toxic event compromising neuronal function through the induction of ROS and impairment of protein clearance machinery (Ciechanover and Brundin, 2003). We tested in your system whether the formation of Tau aggregates could further increase ROS synthesis triggered by rotenone exposure, surprisingly exposure to rotenone in the presence of Tau aggregates did not elicited further increase in ROS synthesis but returned ROS levels to levels similar to control cell cultures.

It is well known that cells can cope with oxidative stress by inducing antioxidant response, to role out whether the formation of Tau aggregates activated antioxidant pathways leading to decrease in ROS synthesis the activity of antioxidant enzymes were measured. The evaluation of antioxidant enzymes activity did not reveal any significant changes associated with rotenone exposure, suggesting that the observed decrease in ROS, protein carbonylation and H_2_O_2_ following Tau aggregation is due to the activation of secondary antioxidant pathways.

Our results raised two interesting observation, (i) that rotenone exposure triggers Tau hyperphosphorylation simultaneously with increases in ROS synthesis, (ii) with the formation of Tau aggregates the initially triggered increase in ROS synthesis is buffered by a pathways independent of changes in the activity of antioxidant enzymes.

Increase of oxidative stress in AD has already been demonstrated with the presence of increased malondialdehyde, 4-hydroxynonenal (Lovell et al., 1995) and protein carbonyls (Kenneth Hensley et al., 1995, Hensley et al., 1996) in brain and cerebrospinal fluid. Growing evidences also show that oxidative stress is increased in Tau transgenic mice (David et al., 2005, Rhein et al., 2009) and that reduction of antioxidant enzymes increase even more Tau hyperphosphorylated levels in these mice (Murakami et al., 2011) suggesting that Tau hyperphosphorylation is possibly linked to dysfunctions in redox homeostasis.

Evidences also have shown up-regulation of Tau kinases as GSK3β and p38 MAPK under oxidative stress conditions in different *in vitro* systems (Goedert et al., 1997, Feng et al., 2013). In your system possibly rotenone effect upon ROS synthesis could be related with its capability to induce Tau hyperphosphorylation, however further experiments need to be done employing antioxidant compounds following rotenone exposure to evaluate this hypothesis.

Protein degradation mechanisms are the first line of defense against the formation of protein aggregates and evidences suggest the disruption of ubiquitin proteasome system (Keller et al., 2000, Carrettiero et al., 2009) and autophagy (Wolfe et al., 2013) in AD. We hypothesized that rotenone effects upon Tau aggregation in your system could be related with dysfunctions in proteasome proteolysis activity or autophagy process possibly triggered by rotenone induced ROS synthesis. We observed a significant decline in proteasomal activity associated with increased oxidative stress parameters following exposure to rotenone in the absence of Tau aggregates. Therefore, following rotenone exposure and in the presence of Tau aggregates we found decreased proteasome activity uncoupled to dysfunctions in oxidative stress parameters. Impairment of proteasome activity as a result of oxidative stress has been shown earlier in HEK and SK-N-MC cells following 100 nM of rotenone exposure (Chou et al., 2010). Our findings partially reinforce this association, since we also found decreased proteasome activity in the absence of oxidative stress.

Proteasome activity is tightly connected to autophagy process and impairment of proteasome activity can induce increase in autophagy process (Ge et al., 2009). In our system we observed notable increase of LC3II levels and autophagolysosomes accumulation following exposure to 0.3 nM of rotenone suggesting autophagy impairment (Mizushima and Yoshimori, 2007) in the absence of protein inclusions, which may be of relevance to trigger protein aggregation (Zhang et al., 2013).

Interesting, our data demonstrate that both autophagy and proteasomal impairment occur before Tau aggregation in your system probably contributing with Tau aggregation at late time points. It has been stated earlier that GSK3β activation by rotenone triggers Tau hyperphosphorylation and also impair of lysosomal acidification possibly reducing autophagy flux (Avrahami et al., 2013). On the contrary, Inhibition of GSK3β is shown to ameliorate autophagic degradation (Parr et al., 2012). From the present study it is evident that rotenone exposure effects could be due to GSK3β activation, suggesting GSK3β as possible target to impair rotenone effects upon Tau dysfunction.

The presence of Tau aggregates associated with increased autophagy flux in your system possibly explaining the lack of ROS dysfunction in the presence of these aggregates. Since evidences suggest that autophagy activation can decrease ROS levels (Filomeni et al., 2015) and increase in ROS synthesis is a potent inducer of autophagy mechanism (Filomeni et al., 2010, Lee et al., 2012).

In rats, although the *in vivo* system may differ from cell cultures, the increase in Tau hyperphosphorylation is induced accompanied by increased hydrogen peroxide levels and a decreased proteasome activity in hippocampus, which corroborates with cell cultures results and indicates that proteasome dysfunction and oxidative stress may be the first signals of cell impairment triggered by rotenone exposure before Tau aggregation.

Together our results highlight the capability of rotenone exposure mimic dysfunction related with the early stages of AD possibly by enhancing ROS synthesis and suggest overall decrease in neuron environment toxicity, measured by protein carbonyls, H_2_O_2_ levels and Tau hyperphosphorylation, following the formation of Tau aggregates.

The decrease in toxicity following Tau aggregation suggest a possible protective effect of protein aggregation mediated by autophagy that could reduce hyperphosphorylated Tau oligomers preventing further cell impairments (Alonso et al., 2006), at least during the temporal window experimentally analyzed in your work. However, it remains to be addressed for how long these protective effects remain active and why they fade allowing the neurodegeneration occurs in late stages of AD and other tauopathies.

## 5 Conclusion

In conclusion this study demonstrated the presence of Tau hyperphosphorylation associated with decrease in proteasome activity and autophagic flux, as well as increase in oxidative stress, precede the formation of Tau aggregates and that during the initial phase of protein aggregation, oxidative parameters seem to return to basal levels revealing a possible protective mechanism, possibly acting through autophagy induction. The cause and effect relation between Tau aggregation, redox homeostasis dysfunctions and alterations in protein degradation pathways following rotenone exposure remain to be elucidated.

## Conflict of interest

The authors declare that they have no conflicts of interest.

## Ethical approval

All applicable international, national, and/or institutional guidelines for the care and use of animals were followed.
